# Natural Selection in Transcription Factor–DNA Interaction Motifs: A Comparative and Population Genomics Perspective

**DOI:** 10.1093/gbe/evaf212

**Published:** 2025-11-12

**Authors:** Manas Joshi, Pablo Duchen, Adamandia Kapopoulou, Stefan Laurent

**Affiliations:** Department of Comparative Developmental Genetics, Max Planck Institute for Plant Breeding Research, Köln, Germany; Institute of Organismic and Molecular Evolution, Johannes Gutenberg University Mainz, Mainz, Germany; Institute of Ecology and Evolution, University of Bern, Bern, Switzerland; Swiss Institute of Bioinformatics, Lausanne, Switzerland; Department of Comparative Developmental Genetics, Max Planck Institute for Plant Breeding Research, Köln, Germany

**Keywords:** regulatory sequences evolution, molecular population genetics, comparative genomics, natural selection

## Abstract

Natural selection heavily influences the evolutionary trajectories of species by impacting their genotype-to-phenotype transitions. On the molecular level, these transitions are shaped by the regulatory sequences. In this study, we employed a combination of population and comparative genomics to investigate how natural selection affects specific regulatory sequence classes involved in the regulatory transcription factor–DNA interactions. These interactions consist of two motifs, namely: transcription factor-binding domains and transcription factor-binding sites. Using publicly available annotation data for *Homo sapiens*, *Arabidopsis thaliana*, and *Drosophila melanogaster*, we first constructed the species-specific lists of the transcription factor-binding domain regions. On applying some of the commonly used summary statistics, we found signals of purifying selection acting on transcription factor-binding domains, consistent with their functional importance. Next, using the biochemical assay-based annotations, we identified potential transcription factor-binding site regions and used variants within them as nonsynonymous equivalents. Interestingly, we also observed that noncoding transcription factor-binding site regions showed similar levels of constraint to that of coding regions for populations with large *N*_e_. Signals of positive selection were limited. Nevertheless, McDonald–Kreitman estimates revealed that, in both fruit-fly and thale-cress, *α* for transcription factor-binding domains was consistently higher than for adjacent nonbinding domains, whereas no such difference was apparent in humans. Taken together, our comparative analysis shows that the efficiency of negative—and to a lesser extent positive—selection on transcription factor–DNA interface elements scales with effective population size. The dataset and analysis pipeline provide a baseline for future studies of regulatory evolution across coding and noncoding regions.

## Graphical Abstract

**Graphical Abstract:**
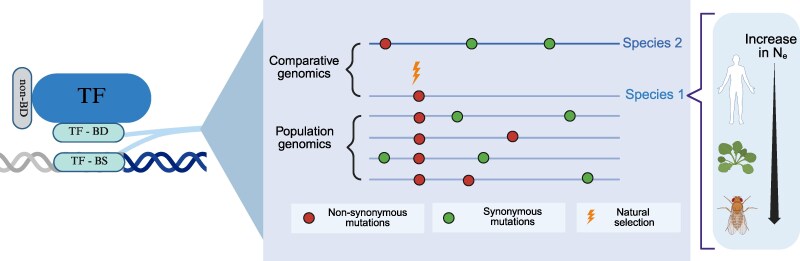

Significance The regulatory sequences (RSs) participating in the regulatory transcription factor–DNA interactions constitute an important class of functional genomic elements that shape the species-specific genotype to phenotype transitions; however, there is still a knowledge gap in understanding the impact of natural selection acting on them. Through a comparative framework, and by using the annotation information on these RSs (embedded within, both, coding and noncoding genomic regions), we primarily noted that the RSs are, overall, under an increased intensity of natural selection than the genomic background and that these signals are highly influenced by the species-specific drift-selection equilibria. This is the first comparative study that observes the impact of natural selection on the said classes of RSs through comparable summary statistics.

## Introduction

Natural selection is an important evolutionary force, and quantifying its genomic impact has been a major focus in the field of evolutionary biology for decades (Ellegren 2008; Vitti et al. 2013). Several studies have highlighted that selection directly affects given phenotypes, which, in turn, contribute to the genetic changes associated with those phenotypes. However, different parts of the genome could be subjected to varying intensities of natural selection. For instance, functional classes like coding regions have been shown to be differentially affected by selection when compared to noncoding regions (Haddrill et al. 2008; Torgerson et al. 2009; Naidoo et al. 2018). The intensity of selection can also vary within the functional coding classes, such as selection acting on synonymous versus nonsynonymous sites (Parsch et al. 2010). Extending such analyses to additional functional categories therefore offers new insights into how genomes evolve.

Transcription factor (TF)–DNA interactions constitute an important class of regulatory interactions that play a vital role in controlling the context-dependent expression of genes, thereby impacting the genotype-to-phenotype transitions. These interactions act as the initial step in the transcriptional process of genes. Given their central role in controlling the expression patterns of the effector genes, these interactions, directly and indirectly, serve various functions ranging from cell differentiation and development (Lee and Young 2013) to controlling multiple biological pathways (Desvergne et al. 2006). These regulatory interactions consist of two interacting motifs: TF-binding domains (TF-BDs) and TF-binding sites (TF-BSs). Here, TF-BDs are stretches of nucleotides within the protein-coding regions of TFs that code for the DNA-binding domains. On the other hand, TF-BSs are stretches of noncoding DNA that are usually located upstream of the effector gene to which the TFs bind via their TF-BD. These interacting motifs are stretches of regulatory sequences (RSs) that play an important role in gene regulation. Genomic variation in either motif can reshape gene expression patterns, but the selective constraints acting on TF-BDs and TF-BSs may differ. Both classes are therefore expected to experience different intensities of natural selection than the genomic background, yet their relative evolutionary pressures remain largely unexplored.

Several studies have highlighted cases of TF-BS regions evolving under the pressure of natural selection when compared to the genomic background (reviewed in [Joshi et al. 2021]). Specifically, TF-BSs have been shown to have a reduced nucleotide diversity as compared to neutral classes, suggesting that these regions are under an increased intensity of purifying selection (Mu et al. 2011; Vernot et al. 2012; Connelly et al. 2013). Interestingly, Vernot et al. (2012) showed that TF-BSs enriched for cell differentiation and development processes showed particularly lower diversity, suggesting that purifying selection acts with varying intensity on different classes of TF-BSs. In addition to the action of purifying selection, studies have also highlighted that these regions are under the influence of positive selection (He et al. 2011; Arbiza et al. 2013). He et al. (2011) also attributed the species-specific gains and losses of TF-BS regions to positive selection. In summary, the evolution of the different classes of TF-BS regions has been documented to be driven by both positive and purifying selection. In contrast, far less is known about the evolutionary dynamics of TF-BDs. Although these coding segments exhibit strong phylogenetic conservation (Romani and Moreno 2021), to our knowledge, no study has yet examined natural selection on TF-BDs through an intra-specific, molecular-population-genetic lens.

In this study, we set out to identify the impact of natural selection acting on both TF-BDs and TF-BSs through a comparative and population genetics framework. To this end, this study spanned three species, namely—*Homo sapiens* (humans), *Arabidopsis thaliana* (thale-cress), and *Drosophila melanogaster* (fruit-fly), and six populations belonging to them (two populations per species). Importantly, the three species have varying magnitudes of effective population sizes (*N*_e_, *H. sapiens* < *A. thaliana* < *D. melanogaster*) and mating systems (outcrossing in *H. sapiens* and *D. melanogaster*; predominantly self-fertilizing in *A. thaliana*). This design lets us view selection against different drift–selection equilibria and over two time-scales: within-species polymorphism and between-species divergence.

For coding regions, we focus on variants that alter TF-BD amino-acid sequences, as such changes can disrupt multiple downstream interactions (Chesmore et al. 2016). For noncoding regions, we compile all variants that fall inside experimentally defined TF-BSs, which likewise have documented functional effects (He et al. 2011; Mu et al. 2011). Across species we detect strong purifying selection on TF-BDs relative to both TF-BSs and control coding regions, whereas TF-BSs show generally weaker constraint—consistent with earlier reports for noncoding DNA (Torgerson et al. 2009; Naidoo et al. 2018). Notably, in the large-*N*_e_ species, constraint on TF-BSs approaches that of some coding classes, hinting that *N*_e_ modulates the strength of selection on regulatory sites. Signals of positive selection are more heterogeneous: several populations exhibit modest evidence of adaptive evolution in TF-BDs, but the pattern is not uniform across all species or populations. Finally, we test the influence of mating system on these inferences with forward simulations under alternative selfing regimes, confirming that reduced recombination in selfers can dampen the detectable footprint of selection.

## Results

### Annotation of the DNA-Binding Domains Within TFs

In this study, we analyzed genetic variation specifically within RSs participating in the TF–DNA interactions across three species: *H. sapiens*, *A. thaliana*, and *D. melanogaster*. For each species, two populations were selected: one representing an ancestral population within the species' original geographic range, and the other expected to have experienced evolutionary pressures associated with colonization of new habitats, such as genetic drift and positive selection. Samples were chosen in a way that minimized the effect of recent admixture or gene flow between the populations. Genome-wide population genomic data were integrated with comprehensive functional annotation data from SwissProt database (Bateman et al. 2017), alongside relevant Gene Ontology (GO) terms. TF-BDs were rigorously identified based on protein domain annotations schema associated with transcriptional regulation and DNA-binding (Fig. S1). This resulted in 886 TFs comprising 1,198 DNA-binding domains for *H. sapiens*, 861 TFs comprising 1,030 DNA-binding domains for *A. thaliana*, and 217 TFs comprising 325 DNA-binding domains for *D. melanogaster*.

To assemble a species-specific catalogue of TF-BS, we began with the ReMap2022 database (Hammal et al. 2022) of cis-regulatory modules (CRMs). High confidence CRMs were defined as those (i) located within ± 2 kb of an annotated protein-coding gene and (ii) carrying an annotation score >30, a metric that marks hotspots bound by multiple transcriptional regulators (TRs). Post-filtering, we retained 22,208 CRMs in humans, 9,736 in thale-cress, and 12,217 in fruit-fly (Table 3). Each retained CRM was then scanned for TR binding using PWM-based TFBS predictions. Because position weight matrices (PWMs) were not available for every TR, motif scanning was restricted to TRs with available PWM information—421 of 1,207 human TRs, 250 of 422 thale cress TRs, and 101 of 550 fruit fly TRs (Table 4). These PWMs were used as input for TEMPLE (Litovchenko and Laurent 2016), which scanned the filtered CRM coordinates at a motif match threshold of *P* ≤ 0.05. The workflow describing the process of identifying TF-BS regions is depicted in Fig. S2. The resulting species-specific TF-BS coordinates, obtained by experimentally informed regulatory regions and TR-specific motif models, constituted the noncoding counterpart to our TF-BD catalogue.

### TF-BDs Under High Selective Constraint at the Population and Species Level

**Fig. 1.**
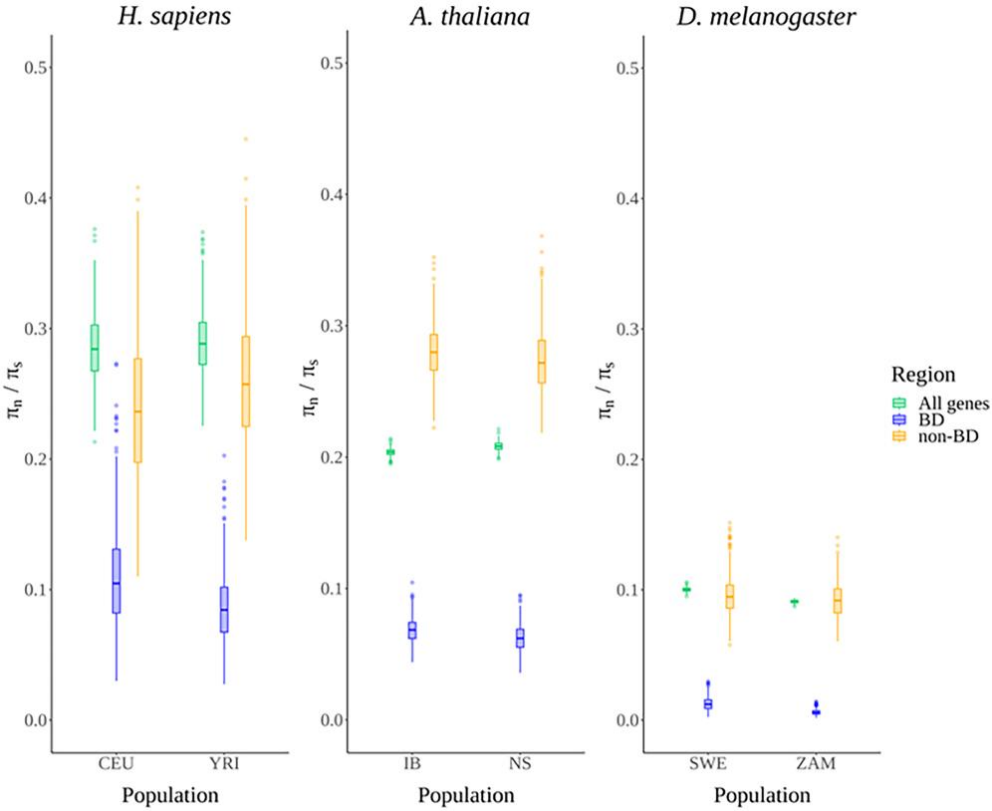
Distribution of π*_n_*/π*_s_* constraint ratios for coding regions, compared across six populations representing three species. Each genomic region class is distinguished by a different color; All genes—all the protein coding genes per species, BD—DNA-binding domains within TFs, and non-BD—functionally unannotated regions within TFs. The distributions were generated through 500 resampling iterations of the constraint ratios to ensure robustness. The population abbreviations are: CEU—Utah residents with central European ancestry, YRI—Yoruba from Ibadan, IB—Iberia, NS—North Sweden, SWE—Sweden, ZAM—Zambia.

We evaluated selective constraint acting on TF-BDs by comparing π*_n_*/π*_s_* ratios across two populations each for three species: *H. sapiens* (CEU and YRI), *A. thaliana* (IB and NS), and *D. melanogaster* (SWE and ZAM) (Fig. 1). π*_n_*/π*_s_* ratios represent the relative variation at nonsynonymous versus synonymous sites, serving as indicators of selective constraint, with lower ratios implying stronger negative selection. Our results showed that TF-BDs consistently exhibited significantly lower π*_n_*/π*_s_* values compared to two control categories: nonbinding domains (non-BDs) of TFs and the entire protein-coding gene set of species (All genes) (Welch's *t*-test, *P*-value < 0.01, Table S1). This pattern persisted across all studied populations and species, reinforcing the functional importance and strong selective constraint acting specifically on DNA-binding domains. Additionally, we observed that selective constraint intensified in species with larger *N*_e_. Specifically, *D. melanogaster* demonstrated the lowest π*_n_*/π*_s_* ratios among the TF-BDs, indicating the comparatively higher efficiency of selection due to reduced genetic drift, while *H. sapiens*, with the smallest *N*_e_, showed the highest π*_n_*/π*_s_* and consequently weaker selection efficiency. Interestingly, non-BD regions generally mirrored All genes, except in *A. thaliana*, where non-BD regions displayed notably higher π*_n_*/π*_s_* ratios, a curious divergence whose biological significance warrants further investigation.

**Fig. 2.**
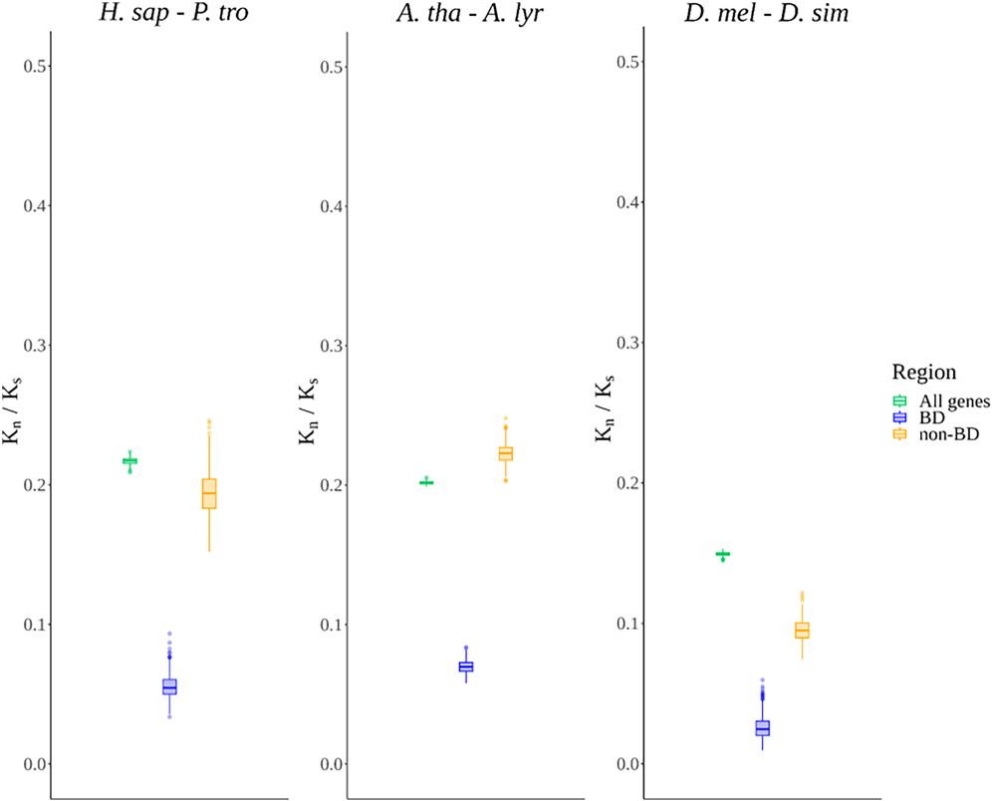
Distribution of *k_n_*/*k_s_* ratios across coding regions, comparing three species with their respective outgroup species (*H. sapiens - P. troglodytes, A. thaliana - A. lyrata, D. melanogaster - D. simulans*). Each class of genomic region is represented by a different color. The distributions were generated through 500 resampling cycles to ensure robustness.

To verify whether the constraint patterns observed at the population level were consistent at the species divergence level, we analyzed constraint ratios (*K_n_*/*K_s_*) derived from pairwise transcript-alignments (**see Materials and Methods**) between *H. sapiens*, *A. thaliana*, and *D. melanogaster*, with their respective sister species, *Pan troglodytes*, *Arabidopsis lyrata*, and *Drosophila simulans* (Fig. 2). Along the lines of the population-level analyses, TF-BDs consistently exhibited significantly lower *K_n_*/*K_s_* ratios compared to non-BD and genomic background regions (Welch's *t*-test, *P*-value < 0.01, Table S2), reaffirming the strong selective constraint on TF-BDs. These analyses, in-line with nearly neutral theory, indicate that the probability of fixation of slightly deleterious mutations increases as effective population size (*N*_e_) declines, i.e. lineages with smaller *N*_e_ exhibit lower selective constraint and an excess of amino-acid altering substitutions, consistent with the fixation of such mutations (Eyre-Walker et al. 2002). In our three focal species, the All genes *K_n_*/*K_s_* follows the expected *N*_e_ gradient (*H. sapiens* < *A. thaliana* < *D. melanogaster*), but for TF-BDs and non-BDs the ordering is distorted because *A. thaliana* shows the highest *K_n_*/*K_s_*—i.e. the weakest constraint—despite having an intermediate *N*_e_.

Our analyses predict strong deleterious effects of mutations occurring in TF-BDs. Hence, we hypothesized that the naturally occurring variants within the TF-BD regions should be enriched for detrimental (pathogenic) variants. For this, we used the *ClinVar* dataset (Landrum et al. 2018), which catalogues variants within the human genome that have been highlighted to have clinical consequences. Specifically, we compared the proportion of the pathogenic variants from the *ClinVar* database (Table S3) in the TF-BD and non-BD classes. We found that variants occurring within TF-BDs are significantly more likely to be detrimental to fitness than those occurring in non-BD regions (Fisher's exact test, *P*-value < 0.01, Fig. S3). This finding further supplements the comparatively high constraint observed on TF-BDs. Specifically, variants within TF-BDs could potentially disrupt multiple gene regulatory interactions, thereby having a detrimental impact on fitness.

### TF-BS Regions Under Relaxed Constraint When Compared to Coding Regions

**Fig. 3.**
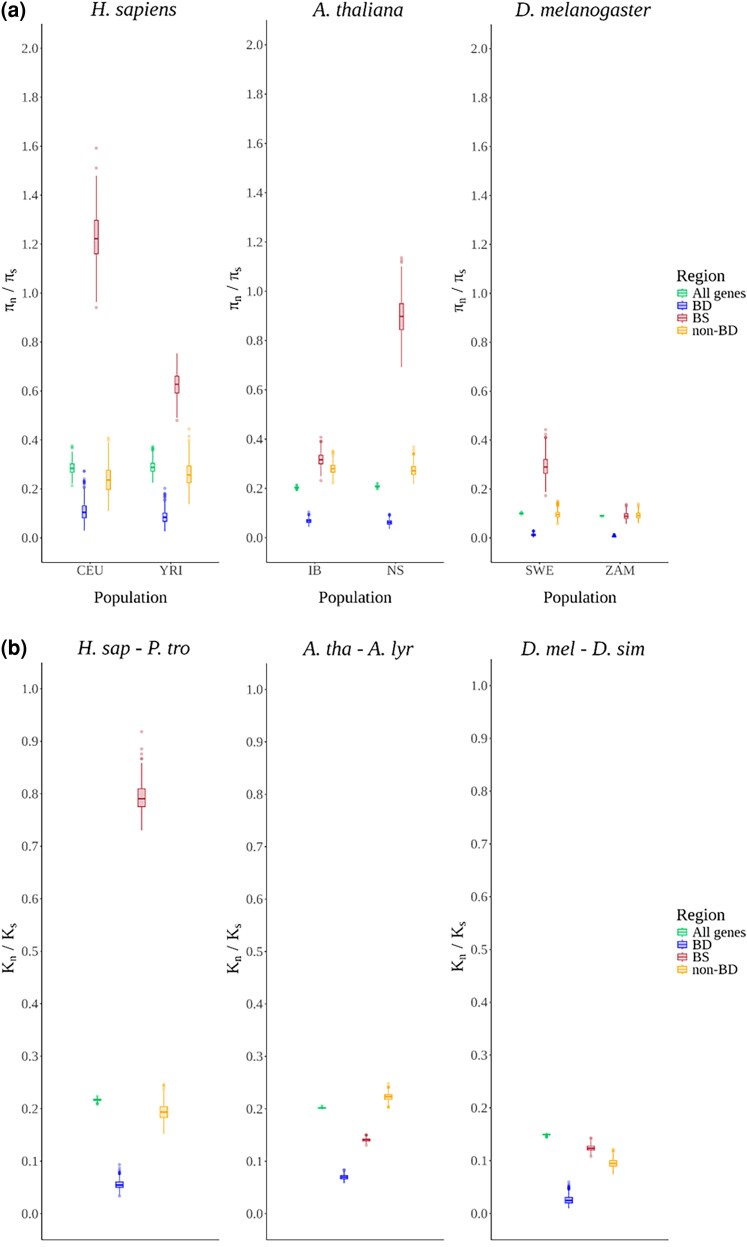
Comparison of constraint ratios between coding and noncoding regions: a) π*_n_*/π*_s_* constraint ratios and b) *k_n_*/*k_s_* constraint ratios for TFBS, TFBD, and two control regions. Distributions were generated through 500 resampling cycles. Different colors represent distinct genomic regions. Population codes are as follows: CEU—Utah residents with Central European ancestry, YRI—Yoruba from Ibadan, IB—Iberia, NS—Northern Sweden, SWE—Sweden, and ZAM—Zambia.

We quantified π*_n_*/π*_s_* at the set of CRM-anchored TF-BSs and contrasted the resulting distributions with those previously obtained for coding regions (TF-BDs, non-BDs, and All genes) across the same six population samples (Fig. 3a). The synonymous class used for TF-BS regions was the synonymous sites within the All genes regions. π*_n_*/π*_s_* ratios for TF-BSs spanned from a minimum of 0.10 in the ancestral *D. melanogaster* population (ZAM) to a maximum of over 1.20 in the derived *H. sapiens* population (CEU), thereby highlighting pronounced among-population variance. The populations with the comparatively lower *N*_e_ also exhibited the largest π*_n_*/π*_s_* at TF-BS (human-YRI and CEU, and thale cress-NS), consistent with less efficient purifying selection in small-*N*_e_ scenarios. Conversely, ancestral-range population of *D. melanogaster* (ZAM) displayed TF-BS ratios that were statistically similar to those measured in the non-BD and All genes regions (Welch's *t*-test, *P*-value > 0.01, Table S1). To validate the signal of selective constraint in noncoding regulatory TF-BS regions, we compared the nucleotide diversity (π) within the IB population of *A. thaliana*. Specifically, we observed that π at CRMs (the π*_n_* component of our TF-BS set) was approximately 3.5-fold lower than in size-matched intergenic regions located outside both coding sequences and CRMs (Fig. S4). This reduction in nucleotide diversity supports the interpretation that the polymorphism screen effectively captured a signal of purifying selection. Finally, in the three colonizing populations (human—CEU, thale-cress—NS, fruit-fly—SWE), π*_n_*/π*_s_* at TF-BS was consistently higher than in their ancestral counterparts, suggesting that recent demographic expansion may have been associated with a reduced efficacy of negative selection and/or facilitated the appearance of adaptive variants segregating at noncoding regulatory sites. Together, these results indicate that TF-BSs are generally subject to purifying selection, but the strength of that constraint is modulated by population history and *N*_e_.

Interestingly, on the divergence scale, TF-BS regions in *A. thaliana* exhibit significantly higher levels of constraint (Fig. 3b) compared to non-BD and All genes regions (Fisher's exact test, *P*-value < 0.01, Table S2). In *D. melanogaster*, TF-BSs show intermediate levels of constraint relative to non-BD and All genes regions. Conversely, in *H. sapiens*, TF-BS regions appear to be under lower constraint compared to all coding regions.

### Comparing Species-Specific Estimates of Adaptive Evolution at Transcription Factors

To measure the intensity of positive selection, we calculated the *α* statistic using the *asymptoticMK* tool (Haller and Messer 2017). In contrast to the traditional MK test, *asymptoticMK* makes use of the SFS information to account for the influence of slightly deleterious mutations in estimating the intensity of positive selection. Estimates of *α* at low-frequency classes are often biased to lower values because these classes typically include an excess of slightly deleterious mutations that segregate transiently but rarely become fixed, inflating polymorphism relative to divergence. For All genes, we observed that the intensity of positive selection, measured by the *α* statistic, was highest in *D. melanogaster*, the species characterized by the largest *N*_e_ and consequently the lowest drift (Fig. S5). This pattern aligns with theoretical expectations since lower levels of drift facilitate the fixation of beneficial mutations, thereby enhancing the overall efficacy of positive selection. However, for the TF-BD and non-BD genomic classes, a high variance in the *α* estimates prevented us from drawing statistically significant observations. The reason for such high variance was the presence of comparatively fewer SNPs in SFS classes for those regions as compared to the set of All genes. While the total number of variants collected in the three species for TF-BD and non-BD regions was not small (Table S4), the *asymptoticMK* method requires, as an intermediate step, that *α* values be calculated for each variant-frequency class independently. Given that the number of variants decreases rapidly with increasing frequency classes, a large sampling variance is associated with most frequency classes in the calculation of *α*, resulting in large confidence intervals (CIs). To address this issue, we used a simpler calculation of *α*, which calculates it directly on the set of variants that have a frequency contained within appropriate frequency boundaries. This cutoff is meant to exclude low-frequency classes, which could potentially be enriched for slightly deleterious nonsynonymous variants.

### Using Customized Frequency Cut-offs per Species for Performing the Traditional MK Test

**Fig. 4.**
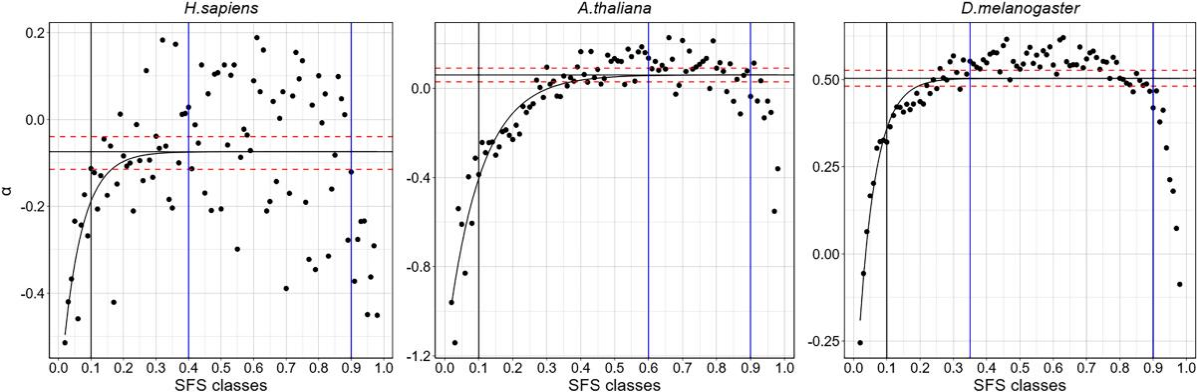
Species-specific asymptote convergence plots to estimate the *α* statistic using the *asymptoticMK* approach for the All genes region. The comparatively older populations per species were chosen as the representative population (*H. sapiens*—YRI, *A. thaliana*—IB, and *D. melanogaster*—ZAM). The SFS classes are indicated on the *x* axis, and the SFS class-specific *α* estimates are indicated as solid black circles. The curved black lines indicate the asymptote, and the convergence point indicates the *α* estimates (CI indicated with dotted horizontal red lines). The high- and low-frequency cutoffs are set by using the convergence of the asymptote as a reference. These cutoffs are indicated per species using solid blue vertical lines and set by visual inspection. The vertical black line is indicative of frequency 0.1, which is often used to remove the influence of slightly deleterious alleles.

To define appropriate frequency cut-off values for each species, we investigated the distribution of frequency-class-specific *α* values for *All genes* region in the ancestral populations produced by asymptoticMK (Fig. 4). Next, we manually defined cut-off values retaining frequency classes for which *α* values stabilized around their asymptote. Low-frequency cutoffs varied for each species. We noted that after reaching a plateau at intermediate frequency classes, *α* values dropped again at high-frequency classes, which led us to exclude these frequency classes too. This is likely due to the well-documented presence of mis-polarized low-frequency variants among the highest frequency classes of the SFS when polarization is done using a single outgroup, as was the case in this study. Hence, high-frequency cutoffs were placed at a frequency of 0.9 for all three species.

**Fig. 5.**
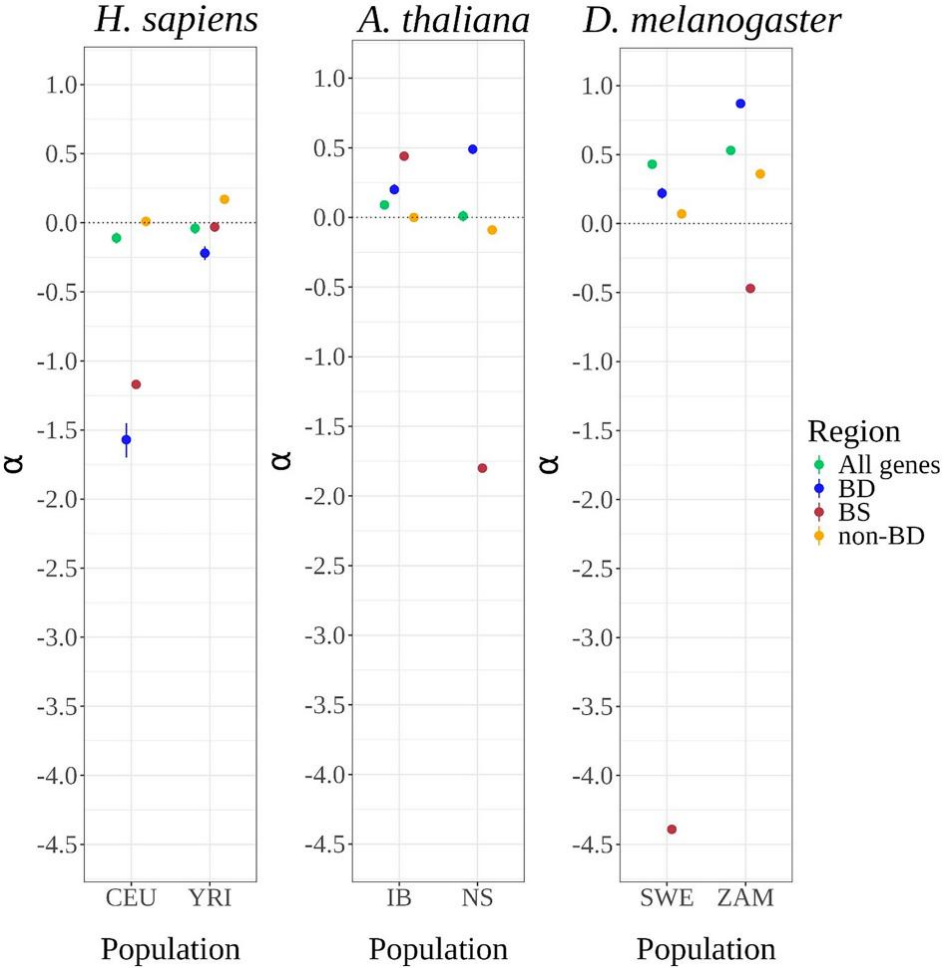
Estimation of region-specific *α* using customized frequency cutoffs. Species-specific frequency cutoffs from Fig. 4 were applied to pool variants, followed by the traditional MK-test. The variance around the estimates is constructed by performing 500 resampling cycles of the polymorphism data. The *α* estimate for the All genes region is calculated using the *asymptoticMK* tool. Colors denote the four genomic regions. Population codes: CEU—Utah residents with central European ancestry, YRI—Yoruba from Ibadan, IB—Iberia, NS—North Sweden, SWE—Sweden, ZAM—Zambia.

Following this procedure, we performed the traditional MK test on the pooled variants to obtain the corrected population-specific *α* values. After applying these steps, we observed that the highest *α* values come from *D. melanogaster* and intermediate values in *A. thaliana* (Fig. 5). Particularly, we noted an overall increase in the *α* estimates for the regulatory TF-BD regions as compared to the other two control regions for species with larger *N*_e_ (*A. thaliana* and *D. melanogaster*). However, this signal fades for species with low *N*_e_ (*H. sapiens*). In the case of TF-BS, we could not detect a strong signal for positive selection, even in populations with large *N*_e_. Except for the IB population, *α* values for the TF-BS regions of all the other populations were negative.

### Self-fertilization Slows the Convergence of Asymptotic *α* in *A. thaliana*

To assess how slightly deleterious variants bias estimates of the proportion of adaptive substitutions (*α*), we plotted frequency-class–specific *α* values for the All genes data set with asymptoticMK (Fig. 4). The curves stabilized earliest in *D. melanogaster*, reaching an asymptote by roughly 0.3 derived-allele frequency. In *H. sapiens*, the plateau was attained later, at about 0.4, while in *A. thaliana* the curve continued to climb until approximately 0.6, implying that mildly deleterious alleles segregate to higher frequencies in this species. Because the three species order *H. sapiens* < *A. thaliana* < *D. melanogaster* in their respective *N*_e_, the especially slow convergence in *A. thaliana* cannot be attributed to drift alone. Instead, it most plausibly reflects this species' predominantly self-fertilizing mating system, which sharply reduces effective recombination (Nordborg 2000). Lower recombination intensifies selective interference among linked sites, allowing slightly deleterious mutations to persist and rise in frequency before being purged by negative selection.

**Fig. 6.**
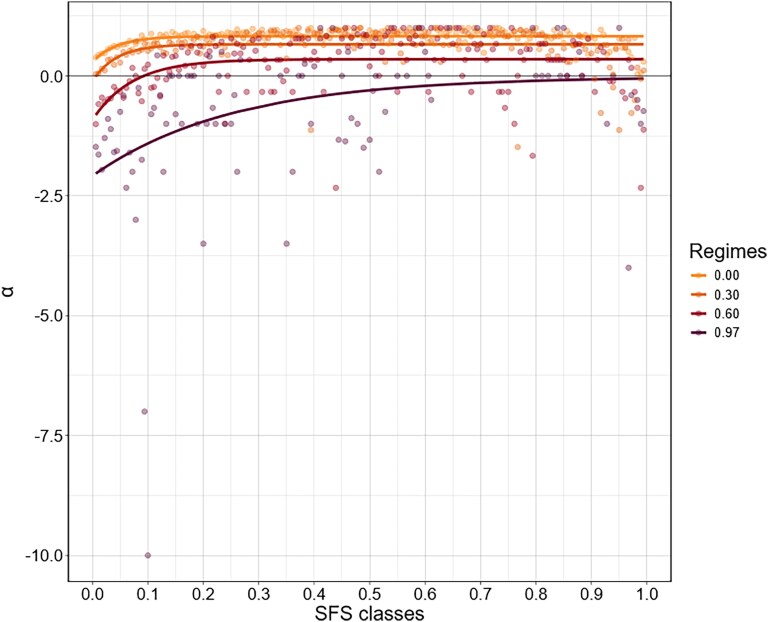
Self-fertilization slows the convergence of asymptotic *α* in forward simulations. Site-frequency-class–specific *α* values were obtained from polymorphism and divergence counts generated with a custom R script applied to data simulated in SFS code (Hernandez and Bateman 2008). Each simulation modeled a 1 Mbp coding region evolving under purifying selection (2*N*_e_*s* = 9) with a mutation rate of 5 × 10⁻^9^ bp⁻^1^, a recombination rate of 1 × 10⁻^8^ bp⁻^1^, and an effective population size *N*_e_ = 2 × 10^5^ (rescaled to 500) over 10^5^ generations. Four selfing regimes are shown: *s* = 0 (outcrossing), 0.3, 0.6, and 0.97 (approximating *A. thaliana*)—plotted as orange, amber, red, and purple curves/points, respectively. Increasing selfing progressively delays the point at which *α* converges on its neutral expectation (zero), indicating that reduced effective recombination under high self-fertilization allows mildly deleterious variants to persist to higher frequencies, consistent with the empirical pattern observed in *A. thaliana*.

To check whether self-fertilization could play a role in the slow convergence of asymptotic *α* in *A. thaliana*, we ran forward simulations with purifying selection with varying selfing regimes (*s* = 0, 0.3, 0.6, 0.97; Fig. 6). As selfing increased, the *α* curves took progressively longer to reach their plateau, eventually producing the same delayed convergence we observe in the empirical *A. thaliana* data for *s* = 0.97 (Strütt et al. 2023). As all other parameters were constant, this pattern likely stems from the reduced effective recombination associated with selfing, which heightens selective interference and allows mildly deleterious variants to persist to higher frequencies. The simulations therefore reinforce our conclusion that high self-fertilization, rather than genetic drift, is mainly responsible for the species-specific delay in asymptotic *α*.

## Discussion

This study focused on elucidating the action of natural selection acting on the regulatory TF–DNA interactions. Given that these interactions are the initiation points for the transcription of the effector genes, this study focused on the two interacting motifs that play a vital role in interactions: TF-BDs and TF-BS. This is the first study investigating the regulatory evolution of both TF-BD and TF-BS elements through a population and comparative genomics framework (but see Joshi et al. 2021). This approach enabled us to observe whether the signals captured on the shorter evolutionary timescales (polymorphism) were robust and consistent on the longer evolutionary timescales (divergence) (Lawrie and Petrov 2014). This study spanned three species, namely, *H. sapiens*, *A. thaliana*, and *D. melanogaster*, and six populations belonging to these species. The interaction domains were carefully identified using the available annotation on TF-BD (Sigrist et al. 2002; Bateman et al. 2023) and TF-BS regions (Jin et al. 2017; Castro-Mondragon et al. 2022). One of the limitations of this study is the conservative filters used to identify these interaction domains, which potentially reduced their number. Notably, the TFs belonging to many of the zinc finger families were omitted due to our chosen annotation schema filters and the segmented nature of their binding domains. Nonetheless, the species-specific TFs are consistently selected using similar filters for all three species. The species-specific TF family distribution is highlighted in Tables S5 to S7.

### Selection Acting on TF-BDs

We found a signal of high constraint acting on the TF-BD regions when compared to the two control classes (non-BD and All genes). This signal was observed to be consistent across all six populations of the three species and both the evolutionary timescales. The high constraint acting on binding domains could be attributed to the functional importance in the context of a gene regulatory network (GRN), which is further supplemented by the high conservation of the TF binding patterns across a broad phylogeny (Nitta et al. 2015). Given the pleiotropic nature of TFs (Chesmore et al. 2016), TF-BDs interact with and control the expression of multiple downstream effector genes. Hence, variants within TF-BDs could disrupt multiple links within GRNs. This signal was further corroborated using clinical variants data from the *ClinVar* database (Landrum et al. 2018). In terms of annotated clinical variants, we found that TF-BDs harbored higher proportions of pathogenic variants when compared to the functionally nonannotated non-BD regions within the TFs, thus confirming the functional importance of the constraint.

### Constructing a Nonsynonymous Equivalent for TF-BSs

Next, we aimed to analyze the signal of constraint acting on TF-BSs. To make the analyses on the TF-BS regions comparable to those of the coding regions, we constructed a nonsynonymous equivalent class for TF-BSs. Specifically, per species, we identified TF-BS regions within the CRMs that were in the vicinity of protein-coding regions. Next, we collected variants occurring within those regions and used these as nonsynonymous equivalents. One of the limiting factors with this approach is not shortlisting variants that are more likely to disrupt the binding affinity. Along the lines of a previous study (He et al. 2011), we attempted exploratory analysis in this direction for the ZAM population of *D. melanogaster*. Specifically, based on the frequency information per-site in the PWM, we formulated a ratio score (Equation 1) metric that scores every variant occurring within the TF-BS region to capture its impact on the binding affinity using the ancestral allele for polarization.


(1) $$\mathrm{ratio}\;\mathrm{score}=\frac{abs(\mathrm{count}\;\mathrm{ancestral}\;\mathrm{allele}-\mathrm{count}\;\mathrm{variant}\;\mathrm{allele})}{\max(\mathrm{count}\;\mathrm{ancestral}\;\mathrm{allele},\;\mathrm{count}\;\mathrm{variant}\;\mathrm{allele})}$$

Using a minimum count cutoff for the frequencies of the ancestral and derived allele (400/1,000), we estimated the π*_n_*/π*_s_* for different ratio score regimes (Fig. S6). It is to be noted that counts here are indicative of the frequency of alleles per position (normalized to 1,000) in the PWM and not the sizes of the studied populations. Overall, we note that the π*_n_*/π*_s_* estimates are similar for the different ratio score regimes, but lowest for the ratio score 0, which consists of all variants pooled together. Surprisingly, we note relatively reduced levels of π*_n_*/π*_s_* with a lenient cut-off scheme and an increase in the retained number of variants. This could result from the comparatively higher nonsynonymous lengths with pooled variants (ratio score 0), which results in lower π*_n_*/π*_s_* (Table S11). Similar trends were also observed for *K_n_*/*K_s_* ratios (Table S12). Since CRMs are densely packed sequences with multiple TF-BSs, more often a single variant overlaps with more than one predicted TF-BS region. Consequently, using the ratio score metric, variants predicted to cause binding alteration for one TF-BS could be neutral/nonaltering for other TF-BS. Hence, adopting this approach was not ideal in our study setup. Therefore, we decided to pool all variants occurring within the annotated TF-BSs.

Next, the rigid cutoffs set for shortlisting the species-specific CRMs that were included in the study could skew our findings. To this end, for the IB population of *A. thaliana*, we estimated π*_n_*/π*_s_* for CRMs that were in the vicinity of the protein-coding regions and having scores of 10 or less (Fig. S7). Here, we noted that the CRM scores tended to have minimal effect on the resulting π*_n_*/π*_s_* estimates.

### Selection Acting on TF-BSs

The TF-BS regions from small *N*_e_ species (such as *H. sapiens*) were observed to be under relaxed constraint. The observation of noncoding elements being under relaxed constraint as compared to the coding elements is in line with several previous studies (Andolfatto 2005; Haddrill et al. 2008; Torgerson et al. 2009; Naidoo et al. 2018). However, interestingly, we found that the levels of constraint on the TF-BS regions were comparable to that of the coding regions for the older populations of large *N*_e_ species (ZAM—*D. melanogaster*). In the case of the ZAM population, the level of constraint on the TF-BS region is similar to the overall protein-coding regions of the genome. These observations suggest that in species with reduced drift, natural selection acts with more efficiency also on noncoding regions. Additionally, on the divergence scale, we could also identify signals of increased constraint on TF-BSs when compared to non-BD and All genes in *A. thaliana*. The TF-BS regions analyzed in this study are located exclusively in the vicinity of the protein coding regions (>99% are in the 2 kb vicinity of All genes for all species), which would also influence the levels of constraint acting on them. Nonetheless, TF-BDs were consistently observed to be under higher constraints when compared to TF-BSs (Fig. 3a and b).

One of the advantages of studying these three species was the differences in their respective effective population sizes (*N*_e_). It has been hypothesized that the efficiency of natural selection increases with an increase in the *N*_e_ due to the reduced influence of genetic drift (Eyre-Walker and Keightley 2007; Galtier 2016; James et al. 2017). The observations indicated that with an increase in *N*_e_, the efficiency of natural selection in removing nonbeneficial and potentially detrimental alleles increases, thereby resulting in reduced π*_n_*/π*_s_* ratios. More precisely, on comparing the π*_n_*/π*_s_* constraint ratios for the four genomic regions (TF-BD, TF-BS, non-BD, and All genes) across the populations of all three species, we found a decrease in π*_n_*/π*_s_* in parallel to an increase in *N*_e_. This observation was in line with the *N*_e_ hypothesis mentioned before. Particularly, we observed lower π*_n_*/π*_s_* ratios for species with high *N*_e_ (*D. melanogaster*) when compared to species with low *N*_e_ (*H. sapiens*). In the case of TF-BS regions, this gradient was even seen for populations within the same species (Fig. 3a). Specifically, we noted a higher π*_n_*/π*_s_* ratio for the populations with comparatively lower drift (YRI—*H. sapiens*, IB—*A. thaliana*, and ZAM—*D. melanogaster*) when compared to populations with comparatively higher drift levels (CEU—*H. sapiens*, NS—*A. thaliana*, and SWE—*D. melanogaster*). We notice however that, in *A. thaliana*, π*_n_*/π*_s_* values in the All genes class were lower than in the non-BD class.

### Quantifying Positive Selection Through *α*

Next, to quantify the action of positive selection, we developed a method derived from the asymptoticMK approach (Haller and Messer 2017) for regions with a small number of genomic variants. We proposed this method to accurately identify the frequency cutoff to limit the influence of slightly deleterious variants segregating within the species. On using *asymptoticMK* for TF-BD and non-BD regions, we noted a high variance around the *α* estimate, which could be explained by the relatively lower number of SNPs per SFS classes. For such regions with fewer variants (TF-BD, TF-BS, and non-BD), we used the convergence point of the asymptote to determine the frequency cutoff per species. On pooling the retained variants, we estimated the intensity of positive selection using the *α* statistic. Here, we found weak signals of high *α* for TF-BD regions when compared to the other classes for high *N*_e_ species (*A. thaliana* and *D. melanogaster*), suggesting positive selection might also act with increased efficiency on TF-BDs. However, these signals were not as decisive as the signals for high constraint on the TF-BD regions. In the case of TF-BSs, we do not find a strong signal of positive selection. One of the factors influencing this outcome could be that the frequency cutoffs used for calculating the *α* statistic for TF-BS were based on the coding region *asymptoticMK* outputs.

In summary, this study probed the impact of natural selection acting on the regulatory motifs involved in the regulatory TF–DNA interactions through a comparative framework. This comparative framework enabled us to make comparisons across multiple species using commonly used summary statistics. The high constraint on TF-BD regions is expected given their functional importance, and this was further corroborated using empirical clinical variant data. Next, using a binding-affinity-based approach, we aimed to compare the impact of natural selection on the noncoding TF-BS regions. We report that TF-BS is under relaxed constraint for small *N*_e_ species; however, their levels of constraint become comparable to that of coding regions for large *N*_e_ species. This finding suggests that with a reduction in drift, selection acts with higher intensity not only on the coding but also on the functional noncoding elements. Finally, the signals for positive selection seemed to overall follow a similar trend to that of negative selection albeit with less intensity. Overall, our findings across all genomic regions are also in concurrence with the overall drift-selection equilibria.

## Materials and Methods

### Divergence and Polymorphism Analysis

For all the three species included in this study, *H. sapiens*, *A. thaliana*, and *D. melanogaster*, we chose the following outgroup species: *P. troglodytes*, *A. lyrata*, and *D. simulans*, respectively (Table 1). To generate alignments of TFs, we performed a gene-based orthology search using the reciprocal-blast approach. Specifically, every representative transcript in the ingroup species was aligned to the entire transcriptome from the outgroup species using *blastn* (Altschul et al. 1990) to obtain an outgroup transcript with the maximum identity score (minimum identity threshold—60%). This outgroup transcript was then aligned back to the transcriptome of the ingroup species to investigate if the initial ingroup transcript had the maximum identity score. Then, the ascertained orthologous pair of transcript sequences from the ingroup and outgroup sequences were re-aligned with *MUSCLE* (Edgar 2004). We also performed polymorphism-based analyses across six different populations from the three species. The species-specific sources of the polymorphism data are listed in Table 1. Polymorphic sites within each population were polarized with the respective outgroup.

**Table 1:** Overview of the polymorphism and divergence data used in this study

Species	Population	Source of polymorphism data	Outgroup species	Source of divergence data
*H. sapiens*	YRI (*n* = 105)	1,000 Genomes (Auton et al. 2015) and (Byrska-Bishop et al. 2022)	*Pan troglodytes* (Mikkelsen et al. 2005)	Ensembl (Cunningham et al. 2022)
	CEU (*n* = 105)			
*A. thaliana*	IB (*n* = 45)	1,001 Genomes (Alonso-Blanco et al. 2016)	*Arabidopsis lyrata* (Hu et al. 2011)	
	NS (*n* = 45)			
*D. melanogaster*	ZAM (*n* = 108)	DGPG3 (Lack et al. 2015) and (Kapopoulou et al. 2020)	*Drosophila simulans* (Clark et al. 2007)	
	SWE (*n* = 14)			

Population codes: YRI, Yoruba; CEU, Utah residents with Central European Ancestry; IB, Iberia; NS, North Sweden; ZAM, Zambia; SWE, Sweden. The population-specific sample sizes are mentioned in brackets.

### TF Annotation and Genomic Regions Included in This Study

We constructed a species-specific list of TFs and the regulatory TF-BDs incorporated within them through annotations retrieved from UniProt (Bateman et al. 2023). To do this, we first extracted all the species-specific manually annotated (*Swissprot identifiers*) protein entries and their respective annotated domains listed on UniProt. We further used domain-specific annotations to retain domains that were annotated with certain GO terms indicating a regulatory role. The regulatory TF-BDs were identified based on the GO terms—“*Transcriptional regulation*,” “*Transcriptional activity*,” and “*DNA binding*” (Fig. S1). Proteins containing at least one domain annotated with the keywords of interest were included in the species-specific TF list. This list of TFs and their corresponding domains was then used in further TF-BD analyses. We then generated a catalogue of the species-specific TF family distribution (by domains) included in this study (Tables S5 to S7). The catalogue of coordinates identified as TF-BD per species is listed in Tables S8 to S10.

Overall, to contrast the signals of selection on the regulatory domains, we constructed two control regions. First, using the domain-specific annotation data per TF, we extracted the functionally unannotated regions within the TF-coding genes. In this study, these regions are referred to as non-BD (nonbinding domains). Given the genomic proximity of the non-BD and TF-BD regions, those two regions would experience similar recombination rates, mutation rates, and GC content. We also used the entire protein-coding gene set per species (which includes the TFs), referred to as All genes, as an additional control region. The All genes class aids in getting an overview of the intensity of selection acting on the overall coding regions of species, and contrasting it to their regulatory elements.

The choice of representative transcripts per gene varied based on the category of genes under investigation. For the TF-centered analyses (BD and non-BD regions), we identified a transcript that had a corresponding manually curated protein identifier in the UniProt database and chose this as the representative transcript. This curated protein entry in UniProt also contained information on the functional domain annotations, thereby enabling us to assign the domain annotation information to every gene. For the All genes analyses, the approach for choosing transcripts varied depending on the species. Specifically, for *H. sapiens*, we chose MANE (Matched Annotation from NCBI and EMBL-EBI [Morales et al. 2022]) transcripts as the representative transcripts. In the case of *A. thaliana* and *D. melanogaster*, we chose Ensembl canonical transcripts as the representative transcripts (Table 2).

**Table 2:** Overview of the species-specific number of genes included in the All genes class and the analysis-dependent choice of representative transcript

Species	No. of All genes	Choice of transcript (BD and non-BD)	Choice of transcript (All genes)
*H. sapiens*	17,751	Uniprot identifier	MANE
*A. thaliana*	27,419	Uniprot identifier	Canonical
*D. melanogaster*	17,679	Uniprot identifier	Canonical

The choice of transcript for the BD and non-BD analysis was exclusively based on the available manually annotated Uniprot (*Swissprot*) identifiers. However, in the case of All genes analysis, this choice was species-dependent. The MANE transcript was chosen as the representative transcript per gene for the *H. sapiens* analysis, whereas for *A. thaliana* and *D. melanogaster* this choice was based on the canonical Ensembl transcript.

### Constraint Ratios

We defined constraint ratios as the proportion of nonsynonymous variants (*test* class variants) to the proportion of synonymous variants (*neutral* class variants). Here, the synonymous variants are used as a control class to counter various confounding factors like change in demography, bottleneck effect, etc. The within-species polymorphism constraint ratios were denoted by π*_n_*/π*_s_*, and the between-species divergence constraint ratios were denoted by *K_n_*/*K_s_*. Additionally, the proportions of nonsynonymous (π*_n_* and *K_n_*) and synonymous (π_s_ and *K_s_*) variants were normalized with their respective Nei–Gojobori distances (Nei and Gojobori 1986). Additionally, to get an estimate of variance around the constraint ratios, we also performed 500 nonparametric resampling cycles to get the CIs around the estimates.

### Access to Clinically Annotated Variants

To calculate the proportions of annotated deleterious variants for TF-BD and non-BD, we employed the ClinVar dataset (Landrum et al. 2018). In a nutshell, the ClinVar database catalogues human genomic variants that are annotated to have clinical significance. The variants were extracted through the ClinVar data repository (date of accession—2021-10-16, see Table S3). These variants were first filtered to retain only SNPs located within the coding regions of the TFs contained in our dataset. These variants were further filtered to retain those having a clinical significance i.e. benign (annotated as—“benign” and “likely benign”) and pathogenic (annotated as—“pathogenic” and “likely pathogenic”). For every genomic region, the proportion of pathogenic variants was calculated by taking the ratio of the number of pathogenic variants to the total number of reported clinical variants (pathogenic and benign).

### Identifying Species-Specific TF-BS Regions and Constructing Nonsynonymous Equivalent

We used the ReMap2022 dataset (Hammal et al. 2022) to identify potential TF-BS regions within the genomes of the three species. Specifically, ReMap2022 catalogues species-specific regions that are binding hotspots for multiple TRs. These regions are identified within the database as CRMs. We used these CRMs as the starting points to identify potential TF-BS regions. We filtered these CRMs to keep those present exclusively in the 2 kb vicinity of the coding regions and having an annotation score of more than 30 (Figs. S8 to S10). This score is reflective of the number of TRs binding within the CRM region. The total number of CRMs accessed from ReMap2022 and the ones retained post-filtering are summarized in Table 3.

**Table 3:** Summary of the CRM information retrieved from ReMap2022

Species	Total CRMs reported in ReMap2022	CRMs retained post-filtering	Minimum overlap threshold within the 2 kb vicinity region
*H. sapiens*	3,329,428	22,208	500 bp
*A. thaliana*	228,624	9,736	500 bp
*D. melanogaster*	591,693	12,217	250 bp

The entire sets of CRMs were filtered to keep a subset which had a score of more than 30 and were in the 2 kb vicinity of the coding regions. The threshold for the overlap with the vicinity is noted in the last column. The shorter overlap length for *D. melanogaster* (250 bp) as compared to *H. sapiens* and *A. thaliana* (500 bp) is explained by the comparatively compact nature of the *D. melanogaster* genome.

In addition to their genomic position, ReMap2022 also provides information on the TRs binding within each CRM. To identify the binding coordinates of each TR, we first retrieved the PWM information per TR from external databases (Table 4). Next, using *TEMPLE* (Litovchenko and Laurent 2016), we scanned for potential TF-BS regions per CRM using the PWM information with a *P*-value cut-off of 0.05 (Fig. S2). Shortlisting potential nonsynonymous variants solely based on the PWM information of individual TRs was challenging. As CRMs are hotspots for binding of multiple TRs, individual variants can have a different impact on the overlapping TF-BS regions. Hence, we pooled all variants identified in the TF-BS regions and used them as nonsynonymous equivalents.

**Table 4:** Summary of the identified TRs within the CRM regions and a subset of them used in this study which had accessible PWM information

Species	The total number of TRs reported to bind within the filtered CRMs	No. of TRs with the available PWM information	Source of PWM information
*H. sapiens*	1,207	421	JASPAR (Castro-Mondragon et al. 2022)
*A. thaliana*	422	250	PlantTFDB (Jin et al. 2017)
*D. melanogaster*	550	101	JASPAR (Castro-Mondragon et al. 2022)

We used the synonymous variants from the coding regions (All genes) as a neutral class for the noncoding TF-BS regions, as in previous studies (Kosakovsky Pond et al. 2005). An important factor for this choice was also that >99% of CRMs per species were in the 2 kb vicinity of the All genes regions. Similar to the coding regions, the obtained proportions of nonsynonymous variants (π*_n_* and *K_n_*) were normalized by their respective Nei–Gojobori distances (Nei and Gojobori 1986). Specifically, the total number of nonsynonymous variants was normalized by the possible number of nonsynonymous sites within the TF-BS regions (entire length of the TF-BS regions).

### Calculation of *α* for the MK Test of Positive Selection

The traditional MK test does not account for the presence of slightly deleterious variants segregating within species when calculating the proportion of adaptive substitutions, *α*. To account for this, tools like *asymptoticMK* (Haller and Messer 2017) look at each mutational class in the site-frequency spectrum (SFS) to make an improved estimate of *α*. Since this method estimates the *α* statistic per SFS class, it is sensitive to the number of variants present per class. Specifically, mainly due to short genomic lengths, the presence of a fewer number of variants in each class could result in inconsistent *α* estimates. Hence, the *α* estimates of comparatively shorter genomic regions (TF-BD, non-BD, and TF-BS) could have high CIs. To account for this, we pooled the variants and employed a combination of the *asymptoticMK* and the traditional MK test. Specifically, we used the SFS class-specific distribution of the *α* estimates for the All genes class per species from *asymptoticMK* to decide on the low and high-frequency cutoffs and keep only the converged *α* values. Such cutoffs were placed by visual inspection on the convergence of the asymptote. This constituted a new dataset, from which we recalculated a single *α* value per genomic region using the traditional MK test. The CIs were constructed by performing 500 resampling cycles on the pooled polymorphism data.

### Simulation Setup for Different Selfing Regimes

To test the effect of selfing on the convergence of *α*, we performed forward simulations using *SFScode* (Hernandez and Bateman 2008). More specifically, we simulated four selfing regimes with selfing rates of 0.0, 0.3, 0.6, and 0.97. Each rate represents the proportion of individuals (in each generation) produced by selfing. Simulations included stretches of 1 Mbp of coding DNA, with a mutation rate of 5e−09 per base pair, a recombination rate of 1e−08 per base pair, and a selective constraint of 2*N*_e_*s* = 9, with *N*_e_ = 200,000 (re-scaled to 500 for the forward simulations). We ran the simulations for 100,000 generations, 45 individuals per population. Each simulation yielded FASTA files from which we used custom R scripts to count the number of synonymous and nonsynonymous changes and calculated *α* for each SFS class and each selfing regime.

### Code Availability

We developed two tools for performing the analyses of the genetic variants occurring within the coding and noncoding regions through a comparative and population genomics framework. *Alag* (https://github.com/Manaswwm/Alag) is tailored for performing the analyses of genetic variants occurring within the coding regions. In this study, we used *Alag* for the analyses of the TF-BD, non-BD, and All genes regions. *templeRun* (https://github.com/Manaswwm/templeRun), a wrapper around TEMPLE (Litovchenko and Laurent 2016), was developed to perform the analyses of genetic variants occurring within the TF-BS regions.

## Supplementary Material

evaf212_Supplementary_Data

## Data Availability

The datasets used in this study were obtained from publicly accessible online databases, which are cited in the relevant sections of the manuscript.
