# Supplementary material for: Natural Selection in Transcription Factor–DNA Interaction Motifs: A Comparative and Population Genomics Perspective
Source: Genome Biol Evol. 2025 Nov 12;17(11):evaf212. doi: 10.1093/gbe/evaf212 (PMC12645836; doi:10.1093/gbe/evaf212)
Supplement: evaf212_Supplementary_Data [file evaf212_supplementary_data.zip › supplementary_figures.docx]

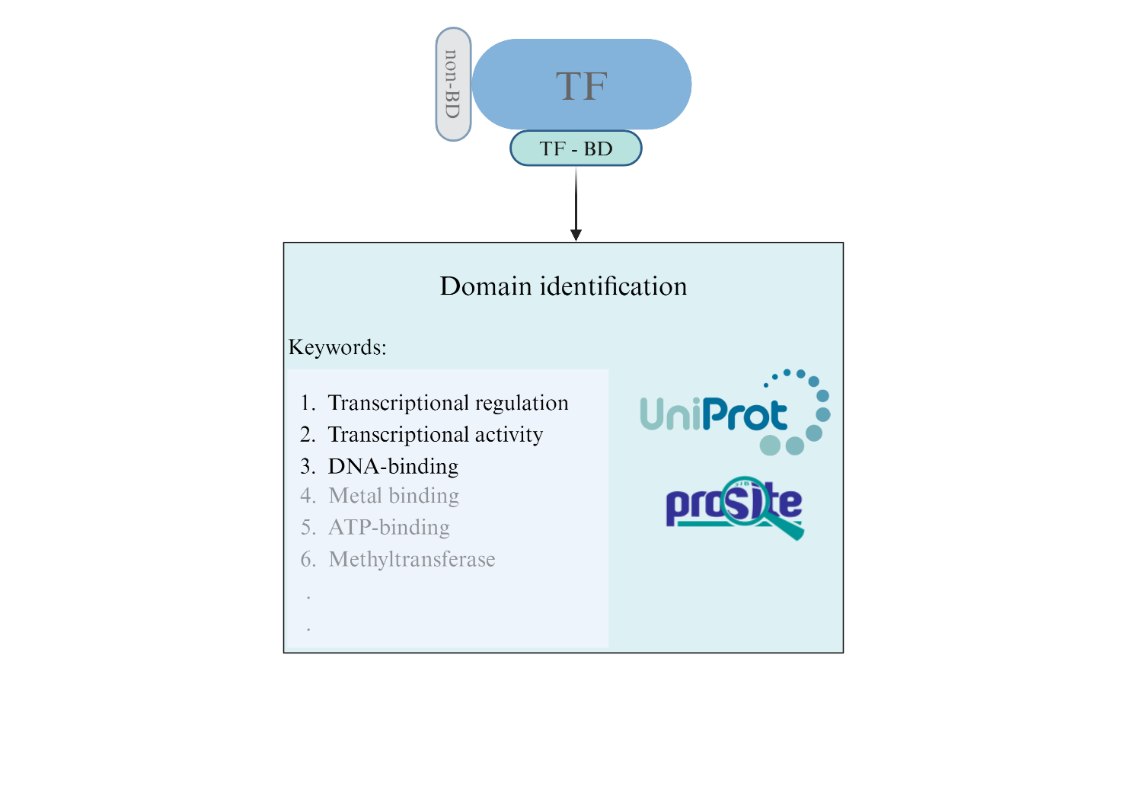


Supplementary Figure 1: Identifying the TF-BD regions using the ontology terms. The annotated domains within the representative UniProt transcript are scanned for the following keywords – “Transcriptional regulation”, “Transcriptional activity” and “DNA-binding”. Domains whose annotations match these criteria are shortlisted as BD.


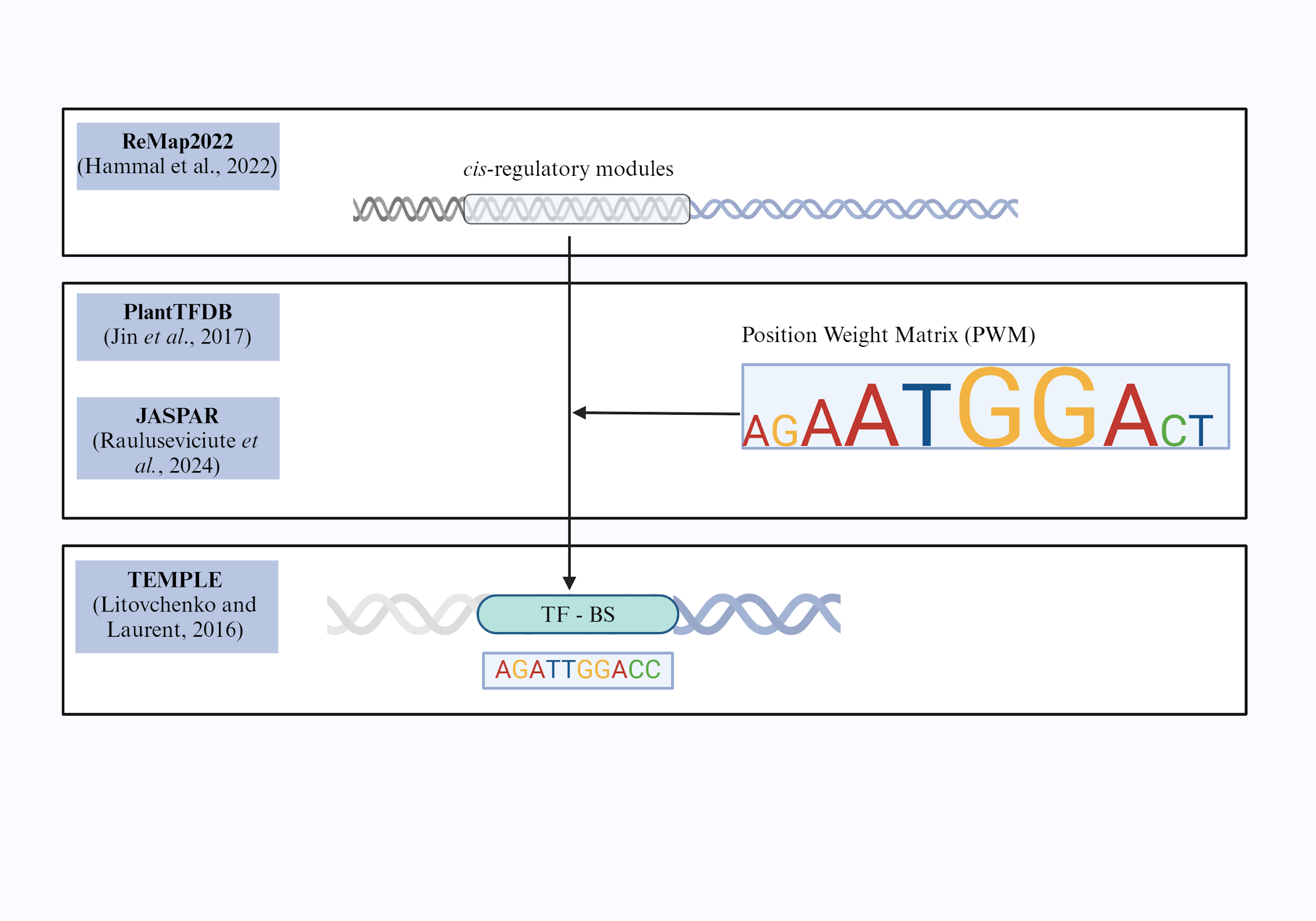


Supplementary Figure 2 – Identifying TF-BS regions and constructing the non-synonymous equivalent class within those regions. (a) – Using ReMap2022 we first extract the species-specific CRM regions, these regions are scanned for specific TF-BS regions by feeding the CRM coordinates and the PWM information of TRs to TEMPLE


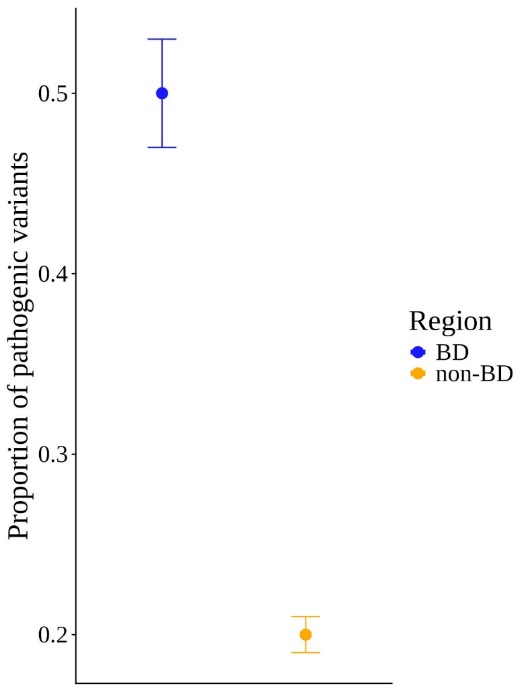


Supplementary Figure 3 - Comparing the proportions of pathogenic variants in the BD and non-BD regions colors indicate the two genomic regions (BD – blue, non-BD – yellow). The proportion of pathogenic variants is calculated by taking the per-region ratio of the total number of annotated pathogenic variants to the total sum of pathogenic and benign variants. The plot indicates the 95% CI with the bootstrapping method and the solid-coloured points indicate the mean.


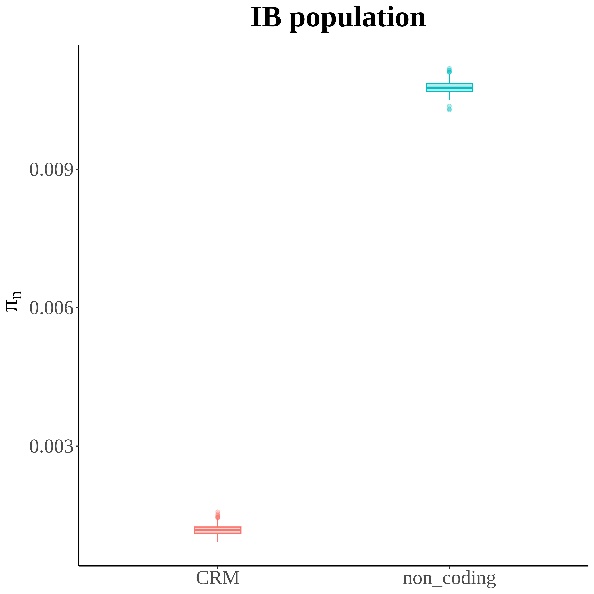


Supplementary Figure 4 - Comparing the nucleotide diversity within the CRM regions (π_n_,CRM) and non-coding & non-CRM (π, non_coding) regions. The non_coding regions represent genomic segments that are neither protein-coding nor annotated as CRMs, and are matched in length to CRM regions. π values for non-coding regions were calculated using a windowed approach.


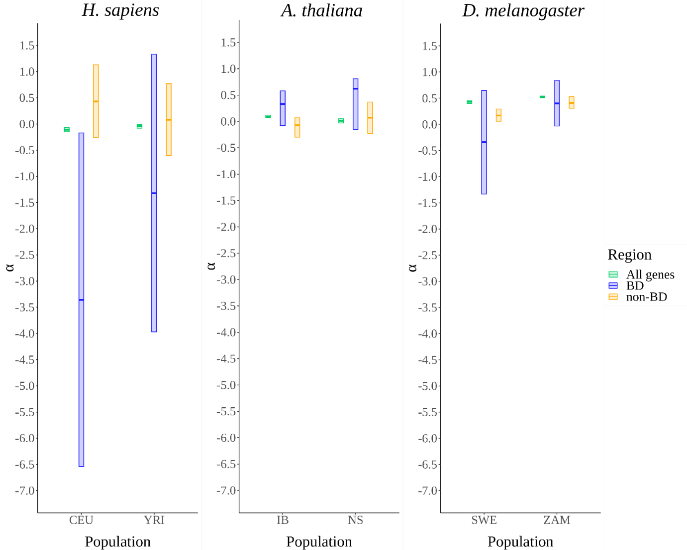


Supplementary Figure 5 – Distribution of the α statistic estimates using the *asymptoticMK* tool for the coding regions across six populations. Colors indicate the three coding regions. The population codes are: CEU – Utah residents with central European ancestry, YRI – Yoruba from Ibadan, IB – Iberia, NS – North Sweden, SWE – Sweden, ZAM – Zambia. Confidence intervals are indicated with boxes and are measure internally by asymptoticMK via bootstrapping


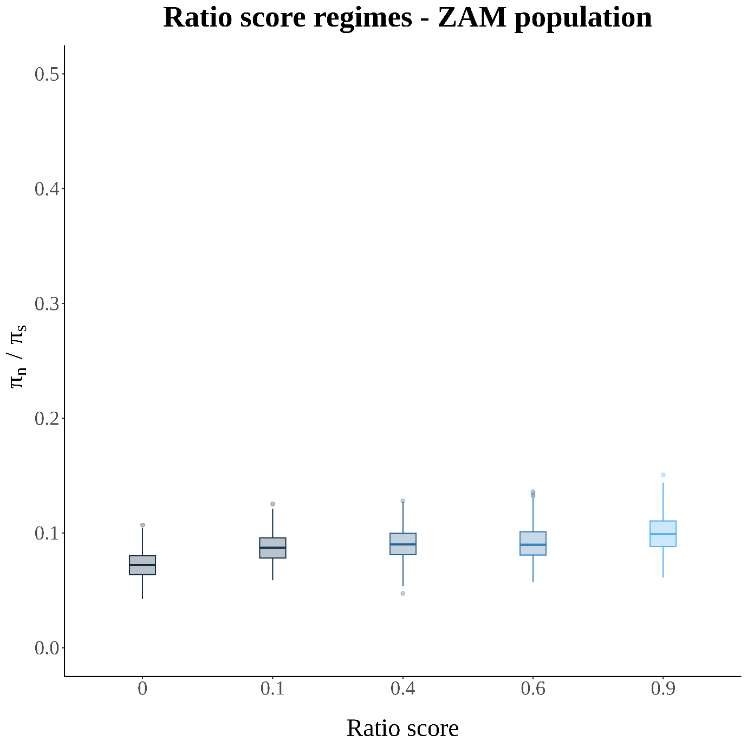


Supplementary Figure 6 – π_n_/π_s_ estimates for the different ratio score regimes in the ZAM population of *D. melanogaster*. Here, π_s_ is consistent across all ratio score regimes and derived from the All genes regions. However, the π_n_ changes depending on the retained variants in the set and the normalization lengths. Specifically, the case of all pooled variants, the normalization length equals the full TFBS length


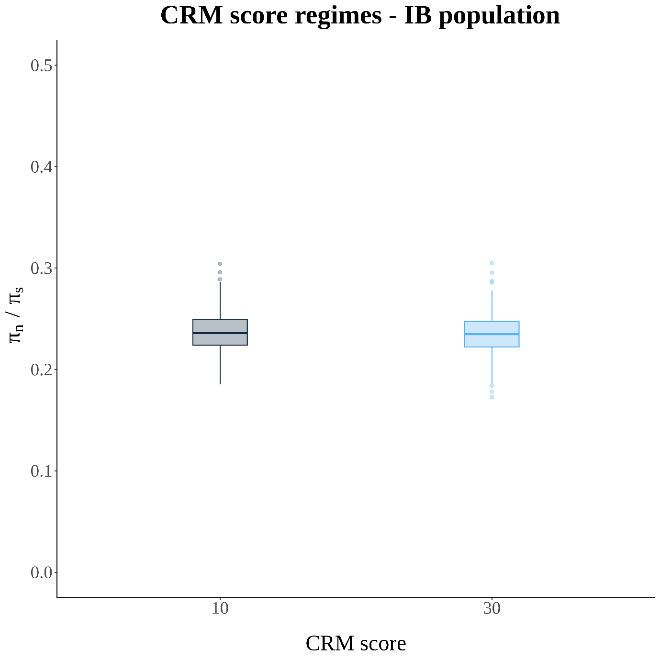


Supplementary Figure 7 – π_n_/π_s_ estimates for the two CRM score based-threshold in the IB population of *A. thaliana*. Score of 10 is reflective of CRMs with score of 10 or less and score of 30 is reflective of CRMs with score of 30 or more


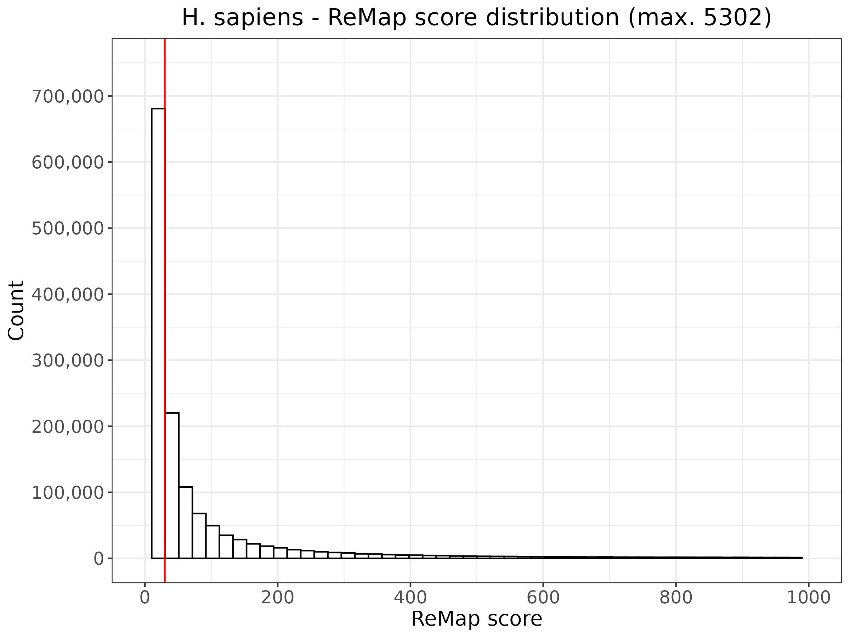


Supplementary Figure 8 – Distribution of ReMap score for CRMs in *H. sapiens*, here scores are associated with the number of TRs binding within each CRMs. The cut-off score of 30 is indicated with the vertical red line. The maximum score assigned to any CRMs is 5302.


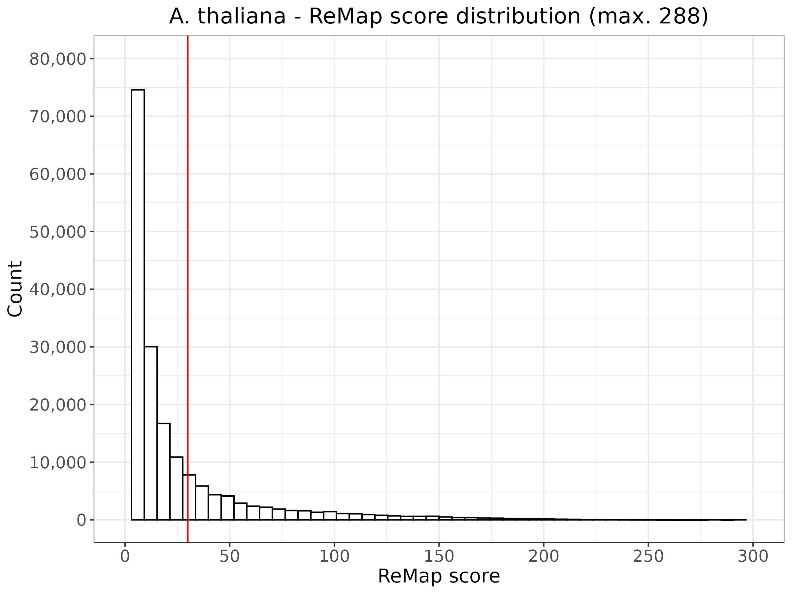


Supplementary Figure 9 – Distribution of ReMap score for CRMs in *A. thaliana*, here scores are associated with the number of TRs binding within each CRMs. The cut-off score of 30 is indicated with the vertical red line. The maximum score assigned to any CRMs is 288.


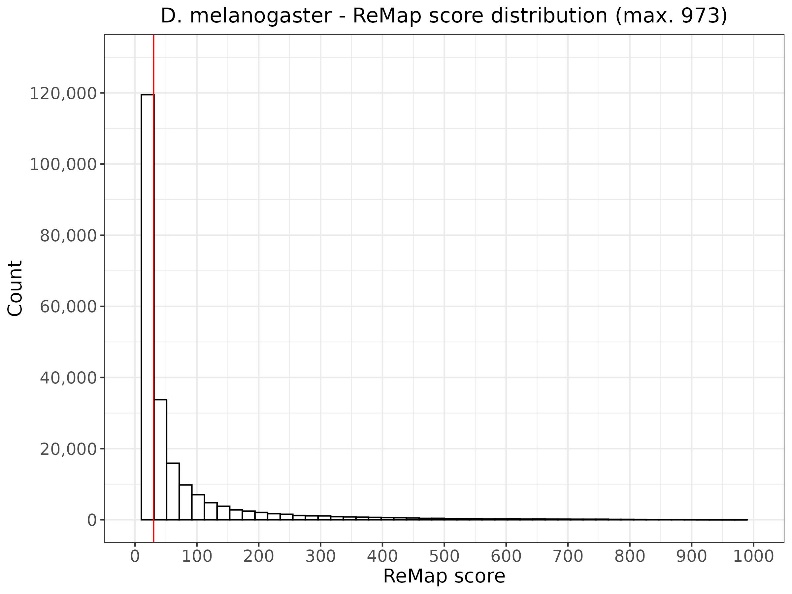


Supplementary Figure 10 – Distribution of ReMap score for CRMs in *D. melanogaster*, here scores are associated with the number of TRs binding within each CRMs. The cut-off score of 30 is indicated with the vertical red line. The maximum score assigned to any CRMs is 973.
